# Oligomannan Prebiotic Attenuates Immunological, Clinical and Behavioral Symptoms in Mouse Model of Inflammatory Bowel Disease

**DOI:** 10.1038/srep34132

**Published:** 2016-09-23

**Authors:** Szilamér Ferenczi, Krisztián Szegi, Zsuzsanna Winkler, Teréz Barna, Krisztina J. Kovács

**Affiliations:** 1 Laboratory of Molecular Neuroendocrinology, Institute of Experimental Medicine, Budapest, Hungary; 2 Department of Genetics and Applied Biochemistry, University of Debrecen, Debrecen, Hungary

## Abstract

Inflammatory bowel disease shows increasing prevalence, however its pathomechanism and treatment is not fully resolved. Prebiotics are non-digestible carbohydrates which might provide an alternative to treat inflammatory conditions in the gut due to their positive effects either on the microbiome or through their direct effect on macrophages and mucosa. To test the protective effects of an oligomannan prebiotic, yeast cell wall mannooligosaccharide (MOS) was administered in dextran-sulphate-sodium (DSS)-induced mouse model of acute colitis. MOS reduced DSS-induced clinical- (weight loss, diarrhea) and histological scores (mucosal damage) as well as sickness-related anxiety. DSS treatment resulted in changes in colon microbiome with selective increase of Coliform bacteria. MOS administration attenuated colitis-related increase of Coliforms, normalized colonic muc2 expression and attenuated local expression of proinflammatory cytokines IL-1a, IL1b, IL6, KC, G-CSF and MCP1 as well as toll-like receptor TLR4 and NLRP3 inflammasome. Some of the protective effects of MOS were likely be mediated directly through local macrophages because MOS dose-dependently inhibited IL-1b and G-CSF induction following *in vitro* DSS challenge and IL1a, IL1b, G-SCF-, and IL6 increases after LPS treatment in mouse macrophage cell line RAW264.7. These results highlight oligomannan prebiotics as therapeutic functional food for testing in clinical trials.

Inflammatory bowel diseases (IBD, Crohn’s disease and ulcerative colitis) are characterized by recurrent inflammation of the gastrointestinal tract affecting increasing number of patients worldwide.

Detailed pathomechanism of IBD is still unresolved, although combination of genetic, environmental and immunological factors has been implicated in the etiology of the disease^1^. Aberrant responses of the innate and adaptive immune system to commensal bacteria are widely implicated in IBD^2^. Classic conservative therapeutic strategies such as anti-inflammatory and/or immunosuppressive therapies, including use of steroids, antibiotics or tumor necrosis factor inhibitors have many serious side effects^3^,^4^ and there is an urgent need for alternative treatments.

Among the animals models of IBD developed so far, the dextran-sodium sulphate –induced colitis has high face validity to the pathogenesis of the human disease^5^. Susceptible strain of mice (C57Bl6) displays clinical and histological signs of gut inflammation which are similar to those seen in IBD patients.

In the normal gut, dendritic cells are continuously monitoring gut microbiota and help to develop tolerance to non-pathogenic bacteria. In IBD patients the epithelial barrier is compromised, allowing commensal bacteria to infiltrate into the lamina propria that triggers local inflammation via release proinflammatory cytokines and propagation of inflammation via recruitment/proliferation of macrophages, neutrophils, and T and B cells. Clinical and experimental observations indicate an imbalance in protective and harmful bacteria in IBD patients. For instance, adherent and intramucosal bacteria are more abundant in patients with Crohn’s disease than in healthy controls^6^. Therefore, manipulation of the gut flora to enhance its protective and beneficial role represents a new promising field to combat IBD. More recently, efforts to manipulate bacterial flora via probiotics or fecal transplants provide an alternative to treat IBD^7^. However, beneficial effects of these treatments in IBD were modest, strain-specific and limited to certain manifestations of disease and duration of use of the probiotic^8^.

As an alternative, the use of prebiotics (non-digestible carbohydrates) is an emerging field to combat IBD. Until now, inulin and fructooligosaccharides (FOS) have been tested in IBD^9^. Based on recent glycomics advances, specific mannose-based oligosaccharides mannooligosaccharides (MOS) can be designed and tested in experimental IBD models for the following reasons: (1) pathogens with mannose specific fimbriae bind to MOS instead of colon epithelial cell surface thus preventing the damage of epithelial barrier and colonization of harmful bacteria^10^; (2) MOS are selectively used by Bifidobacterium and Lactobacilli, thus boosting beneficial members of the gut flora^11^; (3) MOS might neutralize antibodies against mannose epitopes of the yeast Saccharomyces cerevisiae (ASCA)^12^ that have been characterized as serological marker in patients with Crohn’s disease; (4) MOS may act directly on macrophages to regulate cytokine production.

In this study we investigated the effects of a yeast cell wall mannooligosaccharide (MOS) on various markers of DSS-induced colitis in mice. In addition, the direct effect of MOS on murine macrophage cell line (RAW264.7) has also been evaluated.

## Results

### General physiology and sickness behavior

Control mice and MOS exposed animals did not display overt changes during treatment. Starting at day 3, DSS-treated mice showed sickness behavior with decreased locomotion and social withdrawal. At the end of the experiment, 71% of DSS-treated animals have anal prolapse and bloody feces, while only 25% of DSS + MOS treated animals had visible blood in the fecal sample.

### Body weight change

**Figure 1:**
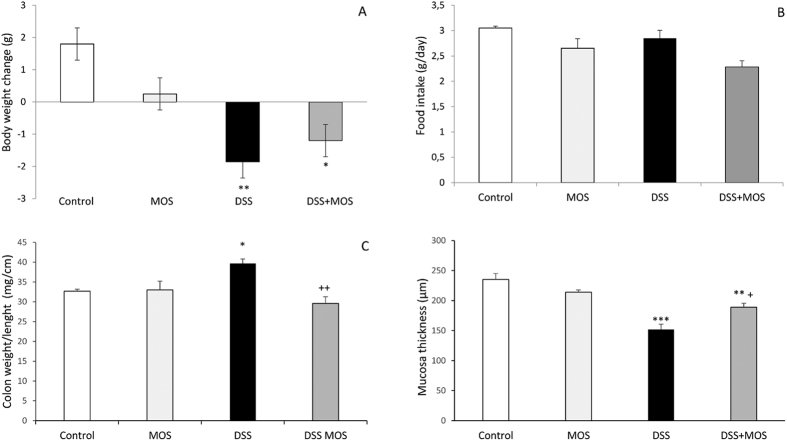
MOS attenuates disease activity in mice during dextran sulphate sodium (DSS)- induced colitis. Mean ± SEM values for body weight gain during 8 days’ treatment (**A**) and daily food intake (**B**). At autopsy the colon weight/length ratio was calculated (**C**), mucosa thickness (**D**) was measured on histological sections. **p < 0.01 and *p < 0.05 compared to control mice; ^++^p < 0.01 and p < 0.05 compared to DSS-treated animals.

There was significant effect of treatment on body weight gain (One way ANOVA, F(3, 18) = 6.69, p = 0.0035). Both DSS and DSS + MOS-treated animals lost significant weight during treatment, while body weight change of MOS-treated mice was not significantly different from that of the controls (Fig. 1a).

### Food and fluid intake

One way ANOVA did not reveal significant difference between the groups in food consumption: F(3, 28) = 1.09, p = 0.37. No significant differences were found in fluid consumption between the groups (F3, 38) = 2.61, p = 0.06 (Fig. 1b).

### Blood glucose

The blood glucose levels (Control: 9.22 ± 0.28; MOS: 9.42 ± 0.31; DSS:8.84 ± 0.36; DSS + MOS: 7.95 ± 0.49 mM) were not different when measured at the time of decapitation F(3, 19) = 2.54, p = 0.092.

### Colon length and weight

One way ANOVA revealed significant treatment effect F(3, 18) = 54.36, p < 0.0001 on colon length. The colon was significantly shorter in DSS-treated mice than those of vehicle treated animals. MOS *per os* administration did not alter DSS effect on colon length. The colon weight/length ratio was significantly higher in DSS-treated animals when compared to control, MOS and DSS + MOS groups (ANOVA F(3, 18) = 7.04, p < 0.0035)(Fig. 1c).

### Histopathology

**Figure 2:**
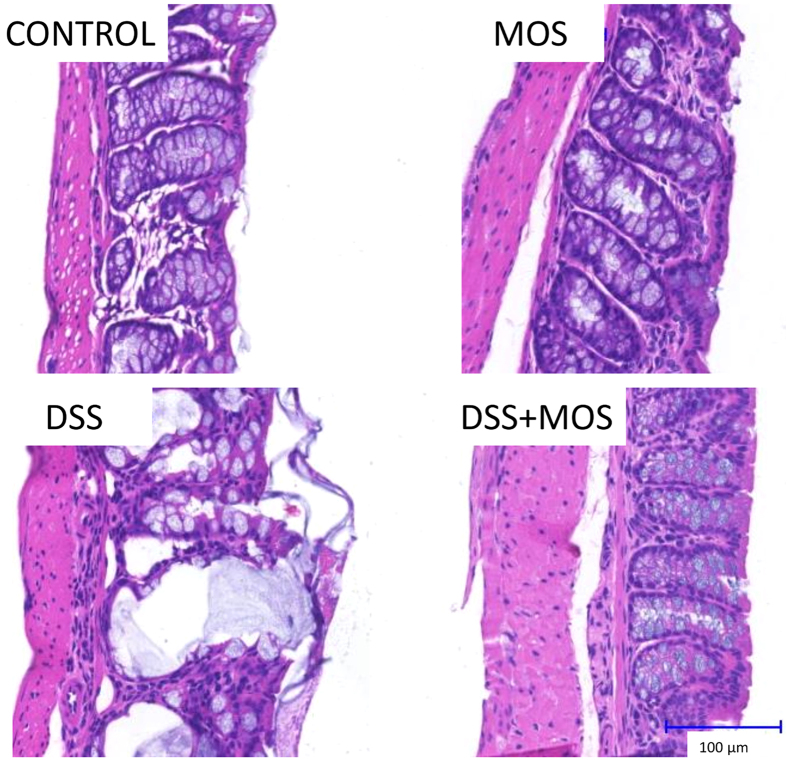
Representative photomicrographs taken from hematoxylin-eosin stained colon sections of control, MOS treated, DSS-treated and DSS + MOS-treated mice. Note the epithelial erosion, disintegrated crypts and piling of leukocytes in DSS-treated animals.

No obvious histopathological abnormality was observed in control mice without challenge. DSS resulted in epithelial damage, decrease of mucosa thickness and crypt disintegration, signs of impaired mucus secretion and invasion of leukocytes into the lamina propria. Histological investigation of the colon in MOS treated animals revealed intact epithelium and increased number of goblet cells. In the DSS + MOS treated group significant goblet hyperplasia with relative intact epithelial layer was seen without leukocyte infiltration. Individual or summarized histopathological scores for inflammation, crypt damage and ulceration indicated less mucosal damage in DSS + MOS treated animals than that of DSS group. Representative examples of H&E stained material are shown on Fig. 2.

### Mucosa thickness and muc2 expression

DSS treatment for 8 days significantly reduced the thickness of the colonic mucosa. MOS alone did not affect mucosal thickness, however, restored DSS-colitis induced reduction of the mucosal layer. (One way ANOVA F(3, 45) = 15.91; p < 0.0001) (Fig. 1d). Relative quantity of Muc2 mRNA was decreased in DSS-treated mice (0.43 ± 0.03) which was normalized by MOS treatment (0.82 ± 0.11).

### Composition of gut microbiome

**Figure 3:**
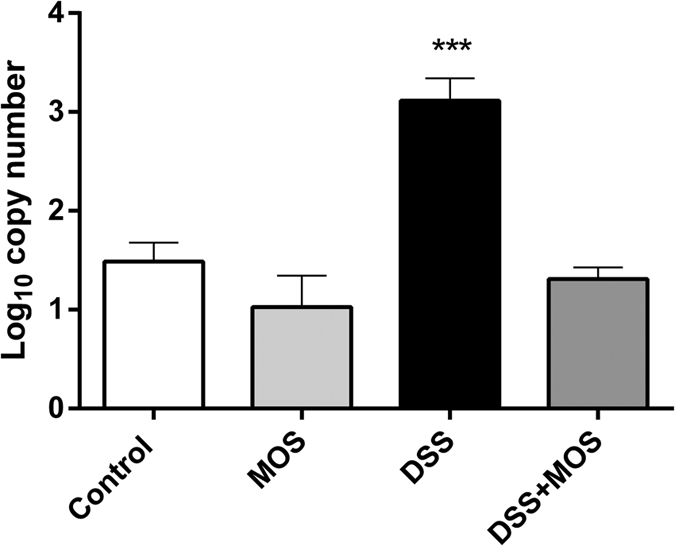
Changes in Coliform bacteria in the colon. Data are expressed as log_10_ copy number/ng genomic DNA isolated from colon content of control, MOS-treated, DSS-treated and DSS + MOS-treated animals. ***p < 0.001 compared to control.

Four main phyla of the mouse gut microbiome (Gram-negative Bacteroidetes and Proteobacteria and Gram-positive Actinobacteria and Firmicutes) have been analyzed from cecum and colon samples using qPCR analysis of phylum-specific 16S rRNA genes. Among these constituents, only Coliforms (Proteobacteria phylum) showed statistically significant change (ANOVA: F(3, 27) = 7.03; p = 0.0015) with an increase in DSS-treated mice (p < 0.01), which was attenuated by MOS treatment (p < 0.01 DSS vs. DSS + MOS). MOS treatment alone had no effect on the quantity of genomic DNA corresponding to Coliform’s 16S rRNA when compared to controls (Fig. 3.)

### Behavior in Open Field Test

**Figure 4:**
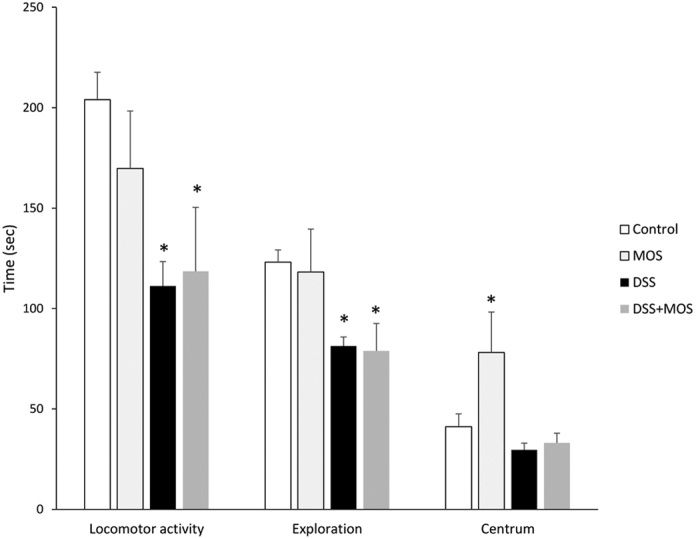
MOS affects mouse anxiety behavior. Effects of MOS and DSS on the locomotor activity, exploratory behavior and time spent in the centrum of open field test. Mean ± SEM values. Mice with DSS-induced colitis display decreased locomotion and exploration, corresponding to sickness behavior. MOS-treated mice are less anxious than control as they spend more time in the open field centrum. *p < 0.05 vs control.

We compared the behavior of mice in open field test, an anxiety-related behavioral paradigm (Fig. 4). There was significant effect of treatment on time spent in the center (One way ANOVA, F(3, 16) = 5.441, p = 0.009). MOS-treated mice spent significantly more time in the centrum of open field apparatus than DSS-treated animals. DSS treatment significantly decreased locomotor activity (One way ANOVA, F(3, 16) = 3.637, p = 0.0357). DSS also decreased exploration (One way ANOVA, F(3, 16) = 3.416, p = 0.0430). In this test there was no difference in horizontal and vertical movement between MOS-treated animals and control mice.

### Cytokine/chemokine mRNA expression in the colon

**Figure 5:**
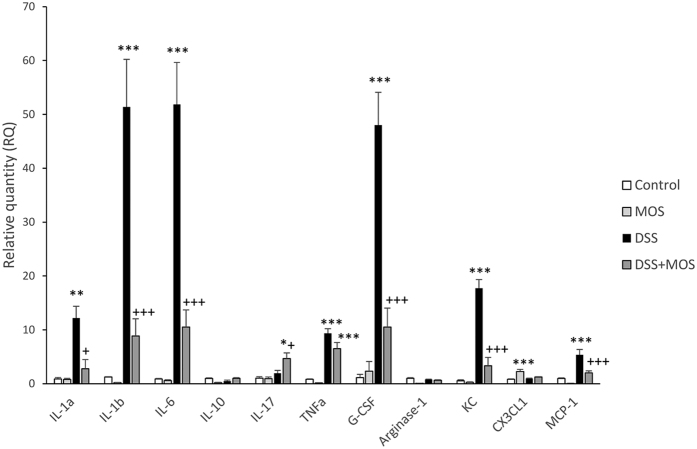
MOS prevents DSS-induced upregulation of select proinflammatory cytokines and chemokines in the colon. Mean ± SEM values of relative mRNA quantities in the colon of mice that were treated with MOS, DSS or DSS + MOS. Cytokine mRNA levels were normalized to GAPDH levels and expressed as relative quantity (RQ) compared to the value of untreated control set to 1. *p < 0.05, **p < 0.01, ***p < 0.001 compared to control mice; ^+^p < 0.05, ^+++^p < 0.001 compared to DSS treated animals.

Because significant piling of leukocytes was seen on histological preparations of DSS-treated animals, which was prevented by MOS, next, we measured mRNA expression of select chemokines and cytokines that are implicated in leukocyte trafficking, recruitment and activation (Fig. 5).

#### Cytokine/chemokine mRNA expression in the colon during DSS-induced colitis

DSS-colitis increased mRNA expression of interleukin IL-1a, IL-1b, IL-6, tumor necrosis factor TNFa, keratinocyte chemoattractant KC/CXCL1, granulocyte colony-stimulating factor, G-CSF and MCP-1. In contrast, no significant changes were found in the expression of IL-10, IL-17, arginase and fractalkine (CX3CL1) in colon samples of DSS-treated mice.

#### Effect of MOS on cytokine/chemokine expression in control mice

Per os treatment of control mice with mannooligosaccharide (MOS) increased the expression of fractalkine and decreased mRNA levels of arginase and KC. The expression of cytokines in the gut remain unchanged.

#### Effect of MOS on DSS-colitis induced cytokine/chemokine expression

MOS treatment significantly reduced DSS-colitis induced IL-1a, IL-1b, IL-6 (F_(3,11)_ = 6.99 p = 0.012), G-CSF, KC and MCP-1 expression. In case of TNFa, however, MOS was ineffective in reducing DSS-induced cytokine mRNA levels.

### Receptors and signal transduction

#### Toll like receptors

**Figure 6:**
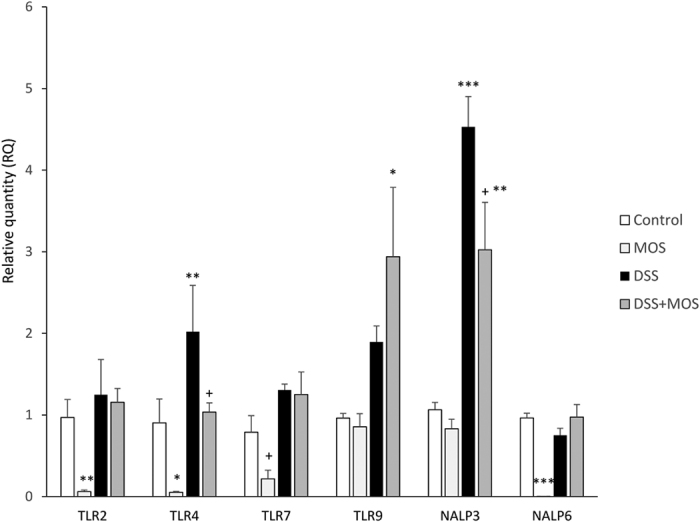
Dextran sulphate sodium and MOS exposure affects Toll-like and NOD-like receptors in the mouse colon. Mean ± SEM values of relative mRNA quantities in the colon samples of mice that were treated with MOS, DSS or DSS + MOS. Receptor mRNA levels were normalized to GAPDH levels and expressed as relative quantity compared to the value of untreated control set to 1. *p < 0.05, **p < 0.01, ***p < 0.001 compared to control mice; ^+^p < 0.05 compared to DSS treated animals.

MOS treatment significantly reduced expression of TLRs 2, 4 and 7, while did not affect TLR9. DSS-treatment for 8 days resulted in elevated mRNA levels of TLR4 and TLR7, while expression of TLR2 and TLR9 did not change in colitis mice (Fig. 6).

#### *Nucleotide-binding oligomerization-domain protein-like receptors* (*NLRs*)

##### NALP3

One way ANOVA revealed significant effect on NALP3 expression: F(3, 11) = 11.34, p = 0.003. Relative expression level of NALP3 was significantly higher in DSS-treated animals compared to controls. RQs were 2.77 ± 0.18 in DSS treated- vs. 1.11 ± 0.11 in vehicle treated controls. Relative quantities values for NALP3 were not different in MOS treated animals when compared to controls. MOS application decreased DSS-induced NALP3 expression to a level, which is not significantly different from control (RQ = 1.10 ± 0.08 in DSS + MOS vs. 1.11 ± 0.11 in vehicle treated controls, p > 0.05).

##### NALP6

8 days treatment with MOS significantly reduced NALP6 mRNA levels in the colon of vehicle-treated control mice F(3, 17) = 27.68, p < 0.001. DSS alone or in combination with MOS did not alter relative quantities of this receptor.

### Plasma cytokine levels

**Figure 7:**
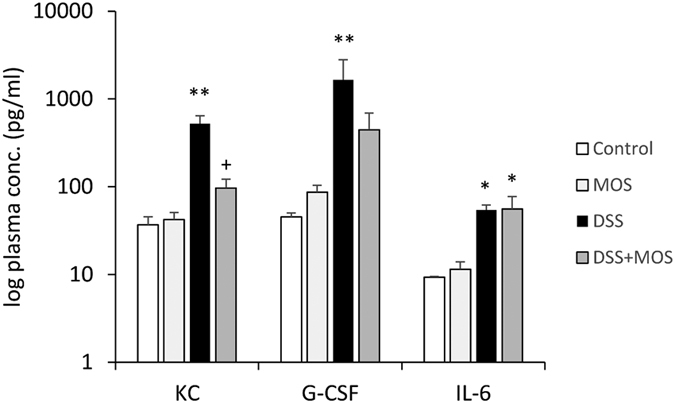
DSS treatment elevates plasma levels of KC, G-CSF and IL-6. Mean ± SEM values of KC, G-CSF and IL-6 in plasma samples of mice that were control or exposed to DSS and treated with vehicle or MOS. *p < 0.05, **p < 0.01 compared to control mice; ^+^p < 0.05 compared to DSS treated animals.

Among the 11 cytokines investigated, cytometric bead array (CBA) did not reveal detectable levels of IL-1a, IL-1b, IL-17a and IL-10 in the control mouse plasma. As analyzed by t-tests, circulating levels of KC (p = 0.0035, t = 3.377 df = 10), G-CSF (p = 0.039, t = 1.961 df = 10) and IL-6 (p = 0.0002, t = 5.211 df = 10) were significantly elevated in mice with DSS-colitis. Plasma levels of most cytokines did not change in control animals treated with MOS, however increased concentration of G-CSF was detected after oligosaccharide administration (p = 0.014, t = 2.875 df = 6) (Fig. 7).

MOS treatment successfully prevented DSS-induced KC levels (One way ANOVA: F(3, 18) = 7.87, p = 0.002). In case of G-CSF there was a tendency for reduction by oligosaccharide, however, the difference between DSS and DSS + MOS groups did not reach significance due to high individual variation. Elevated plasma levels of IL-6 seen in DSS colitis group was not reduced by MOS.

### *In vitro* effects of MOS on RAW 264.7 macrophage cells

**Figure 8:**
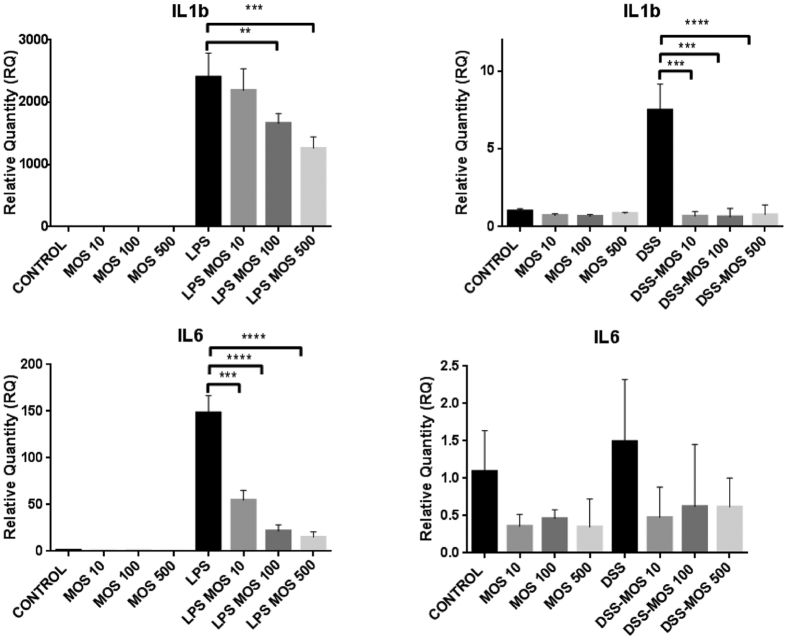
MOS prevents LPS or DSS-induced cytokines in RAW 264.7 mouse macrophage cell line. Mean ± SEM values of relative mRNA quantities measured in RAW 264.7 macrophages following DSS (5 μg/ml) or LPS (100 ng/ml) and 0, 10, 100, 500 μg/ml MOS administration. **p < 0.01, ***p < 0.001, ****p < 0.0001.

To assess the direct effect of MOS on macrophages, a murine macrophage cell line, RAW 264.7 was treated with MOS alone and in combination with DSS or LPS. MOS alone does not induce expression of IL-1a, IL-1b, TNFa or KC, however, 100 μg/ml dose significantly elevated the mRNA level of G-CSF. By contrast the RQ levels of IL-6 mRNA were reduced in all MOS treated cells, however the decrease was not significant. LPS as an inflammatory challenge, resulted in significant elevation of all cytokine expression except KC. LPS-induced proinflammatory cytokine mRNA levels were significantly and dose-dependently reduced by MOS except TNFa. DSS treatment selectively increased the expression of IL1b and G-CSF and this elevation was completely prevented by MOS administration (Fig. 8).

## Discussion

Here we have provided evidence that yeast mannooligosaccharide (MOS) with beta 1,4 linkage is effective to relive symptoms of inflammatory bowel disease by reducing colitis-related increase of Coliform bacteria, expression proinflammatory mediators and boosting mucus production in the sodium dextran sulphate-induced colitis model in mice.

### Methodical considerations

Sodium dextran sulphate (DSS)-induced colitis is extensively used to model inflammatory bowel disease and screen therapeutic interventions in rodents^13^,^14^. Our experiments were performed in C57Bl6 mouse strain, which is more susceptible to DSS than other strains such as CD1 or BalbC^15^,^16^. Colitis was induced by addition of DSS to drinking water. As the fluid intake was not different among the different treatment groups, the beneficial effect of MOS may not due to differences in DSS-water consumption. Our experimental design (8 day oral DSS exposure) corresponds to an active colitis and recapitulates many clinical features of human ulcerative colitis (UC)^14^.

### DSS-induced colon inflammation

We have confirmed significant pathological- (body weight loss, diarrhea, reduced colon length), and histological- (reduced mucosa thickness and epithelial damage) alterations in DSS- treated mice^17^,^18^. Although the mechanism with which DSS induces colonic inflammation in mice is not fully clear, it is generally held that DSS is toxic to gut epithelia, induces erosions and compromises gut barrier^19^. Impaired barrier functions (leaky gut) allow the invasion of luminal bacteria and induce local innate immune response at the gut associated lymphoid tissue.

IBD is manifest in the context of commensal microbes. Studies of experimental animal models of IBD reveal that germ-free mice normally display only a few signs of inflammation and for the full effects of experimental colitis exposure to natural microbial communities is necessary. Although there is still a considerable controversy whether the entire commensal microbiota or individual pathogens (e.g. *Mycobacterium avium* spp. *paratuberculosis*) are primarily responsible for induction of inflammation. Colonic disbiosis have been implicated in the pathogenesis of human IBDs^20^, with a decrease of relative abundance of Enterobacteria and Firmicutes and an increase of Proteobacteria and Actinobacteria in case of UC^21^. Furthermore, the numbers of Enterobacteriaceae in colonic biopsies of IBD patients were 3–4 logs higher than in the controls^22^. Here we also show a selective increase of Coliform bacteria in the colon and cecum content of DSS-treated mice, which confirms similarities between DSS mouse model and human UC. These Gram negative bacteria, which are recognized as pathogen signals by toll-like receptor-expressing cells of the innate immunity to induce inflammation via release of various proinflammatory mediators^23^,^24^. Furthermore, colonic inflammation is accompanied by significant recruitment of leukocytes to the submucosa. Along these lines, elevated expression of various chemo-attractants, such as monocyte chemotactic protein 1 (MCP-1, CCL-2), keratinocyte chemoattractant (KC or CXCL1) and fractalkine (CX3CL1) has been detected in the colon of DSS-treated animals corresponding to recruitment of neutrophils, monocytes and macrophages, respectively^25^,^26^,^27^. It has been recently shown that DSS is associated with medium-chain-length fatty acids (MCFAs) to form nanometer sized particles, which may act as intracellular danger associated molecular patterns (DAMPs) in the colonic mucosa together with luminal bacteria (PAMPs) to trigger inflammatory pathways^28^. Indeed, we have detected significant upregulation of toll like receptors (TLR 4, TLR 7 and TLR 9) as well as NOD-like receptors whose activation ultimately results in synthesis and release of proinflammatory cytokines, IL-1a, IL-1b, IL-6 and TNFa in the colon of DSS-treated mice. The inflammasome NLRP3 plays a crucial role in the pathogenesis of colitis as shown recently in NLRP−/− mice^18^. Our present results confirm these findings by revealing elevated NALP3 mRNA levels in the inflamed colon. By contrast, according to Zaki *et al*. NLRP3 inflammasome protects against epithelial injury in DSS colitis^29^.

### Protective effects of MOS in DSS-induced colitis

When MOS was given to DSS-treated mice it prevented and/or significantly attenuated colitis symptoms although MOS administration in control animals did not result in overt changes in physiological parameters or clinical scores. The beneficial effects of various prebiotics, such as oligofructose and inulin on gastrointestinal and immune homeostasis have already been suggested^9^,^30^,^31^,^32^,^33^, however, the effects of mannooligosaccharides have not been fully addressed. From our present data it is very likely that MOS targets different loci in colitis pathomechanism. For instance, MOS is able to capture pathogenic bacteria through direct interaction with specific receptors, has a protective effect through stimulation of mucus production^34^ and it is very likely that MOS has a direct effect on gut associated lymphoid tissue (GALT) i.e. on macrophages^35^.

There is compelling evidence for binding of MOS to FimH, a mannose specific lectin expressed in Gram negative bacteria with Type1 fimbriae, such as Salmonella^36^ or pathogenic E. coli^37^,^38^. This competitive interaction significantly reduce bacterial adherence to intestinal epithelial cells bearing mannose-containing glycoprotein receptors. Our present data revealed that MOS attenuated the increase of Coliform bacteria in the DSS- treated mouse colon. Furthermore, the presence of MOS likely serves to opsonize the bacteria by attracting mannan-binding lectin (MBL), a normal acute phase protein considered a part of the lectin pathway of the innate immune system, leading to opsonophagocytosis of the bacteria.

Previous research revealed interactions of different oligosaccharides (MOS, FOS and inulin) with carbohydrate receptors (mannose receptors, galectin family receptors, and Toll-like receptors) located on either epithelial or immune cells in the gastrointestinal tract^30^,^39^. For instance, a double-blind placebo controlled human study on ulcerative colitis patients revealed significant immunomodulatory effect of a symbiotic containing FOS and inulin with reduced levels of beta defensins, IL-1 and TNFa mRNA and improvement of the full clinical appearance of chronic inflammation^40^. Our results on RAW264.7 cells suggest that MOS has a direct effect on macrophages by preventing LPS or DSS-induced IL-1b and GCSF mRNA.

One intriguing finding of the present study is that DSS treatment and MOS administration affected the behavior of the animals. MOS in control animals resulted in decreased anxiety and increased exploration. By contrast, DSS-treated animals showed sickness behavior with significant reduction of locomotor activity and signs of increased anxiety (more time spent in the periphery of the open field), which was not affected by MOS administration. Indeed, it has been shown that anxiety and depression are common psychiatric symptoms in patients with chronic gut disorders, including those with overt inflammatory conditions of the gastrointestinal tract, such as inflammatory bowel disease. Although the mechanisms of gut-brain interactions are not fully described, inflammatory mediators (i.e. IL-1), which are released in the gut are able to affect directly or indirectly the central nervous system functions^41^.

Taken together, our results highlight protective effect of yeast cell wall mannooligosaccharide (MOS) on clinical-, histological and behavioral symptoms in the mouse model of inflammatory bowel disease. MOS acts directly on macrophages to relive production of proinflammatory mediators and indirectly by attenuating bacterial burden and via increased mucin expression. These findings identify MOS as a potential functional food ingredient for human clinical trials.

## Materials and Methods

### Animals

Adult (8–10 weeks old) male C57Bl6/J mice were obtained from the local colony bred at the Medical Gene Technology Unit (Specific Pathogen Free, SPF level) at the Institute of Experimental Medicine Budapest Hungary. Animals were housed at the Minimal Disease (MD) level, 3–5/cage under controlled environmental conditions: temperature, 21 °C ± 1 °C; humidity, 65%; light-dark cycle, 12-h light/12-h dark cycle, lights on at 07:00. Mice had free access to rodent food and water. All procedures were conducted in accordance with the guidelines set by the European Communities Council (86/609/EEC/2 and 2010/63 Directives of European Community) and the protocol was approved by the Institutional Animal Care and Use Committee of the Institute of Experimental Medicine, Budapest Hungary (permit numbers: 22.1/3347/003/2007 and PEI/001/29-4/2013).

### Materials

Dextran sulphate sodium (DSS) was purchased form (MP Biomedicals, Santa Ana, CA, USA; cat #160110, Mw: 36000–50000). MOS was obtained from Alltech (Kentucky, USA).

### Experimental Procedure

Animals were handled for 3–5 days to get acquainted with oral gavage using water only. Four experimental groups were then formed: (1) control, (2) yeast cell wall mannooligosaccharide (MOS) treated; (3) dextran sulphate (DSS)-treated and (4) DSS + MOS treated animals. Mice in groups 1 and 2 received tap water to drink while animals in groups 3 and 4 had 3.5% (w/v) DSS to drink. MOS or vehicle (water) was given through oral gavage. Both DSS and MOS treatments started at the same time and lasted for 8 days. Mannooligosaccharide was dissolved in drinking water and applied at the dose of 500 mg/kg bw) which was selected on the basis of our pilot dose-response study (100–500–1000 mg/kg/day) where the clinical scores were evaluated only.

Body weight of the animals has been measured every day during treatment. Physiological status, general behavior of the animals and stool consistency were also recorded on a daily basis.

### Behavior analysis

On the last treatment day (before dosing MOS/vehicle), behavior of the animals was tested in open field (white plastic box 40 × 40 × 30 cm). Mice were placed in the center of the box and allowed to explore the apparatus for 5 min. Behavior was recorded by video camera and analyzed by H77 computer based event recorder-program (Jozsef Haller, Institute of Experimental Medicine, Budapest, Hungary). Locomotor activity was calculated from total horizontal and vertical movement. Central area exploration was also recorded to provide an additional measure for anxiety.

At the end of the treatments, mice were quickly decapitated trunk blood collected in ice cold EDTA containing plastic tubes, centrifuged and the plasma stored at −20 °C until assay. After decapitation, a drop of blood was applied to the D-Cont Personal blood glucose meter (77 Elektronika LTD Budapest, Hungary) for blood glucose measurement.

Cecum and colon was exposed by midline laparotomy, the length and weight of the colon (after feces removal by flushing with PBS) was measured and processed for biochemical and histological evaluation.

### Histology

Tissue samples were fixed in 10% formalin and embedded in paraffin. 5 μm sections were cut, stained with hematoxylin-eosin, scanned by tissue scanner (3D Histech Budapest, Hungary) and evaluated by Pannoramic Viewer 1.15 program (3D Histech, Budapest, Hungary).

### Cell culture

Murine macrophage cell line, RAW 264.7 was purchased from American Type Culture Collection (ATCC, Manassas, VA, USA) and cultured in DMEM supplemented with 10% heat inactivated fetal calf serum and antibiotics (100 units/mL of penicillin and 100 μg/mL of streptomycin). 5 × 10^4^ cell were plated onto 24 well plastic culture dishes and treated with MOS (10, 100 and 500 μg/ml) for 2 hours and then challenged either with DSS (5 μg/ml) or LPS (100 ng/ml) for 24 h. Supernatants were collected, cells washed and 500 μl TriSol reagent was added to each well.

### Real time qPCR measurements

For quantitative real time PCR measurements, tissue samples were cut and immediately frozen in dry ice. Total RNA was isolated from colonic and cell culture samples with QIAGEN RNeasy Mini Kit (Qiagen, Valencia, CA, USA) according the manufacturer’s instruction. To eliminate genomic DNA contamination DNase I treatment was used (100 μl RNase-free DNase I (1 U DNase I, Fermentas) solution was added). Sample quality control and the quantitative analysis were carried out by NanoDrop (Thermo Scientific). Amplification was not detected in the reverse transcription (RT)-minus controls. cDNA synthesis was performed with the High Capacity cDNA Reverse Transcription Kit (Applied Biosystems, Foster City, CA, USA). The designed primers (Invitrogen) were used in the Real-Time PCR reaction with Power SYBR Green PCR master mix (Applied Biosystems, Foster City, CA, USA) on ABI StepOnePlus instrument. The gene expression was analyzed by ABI StepOne 2.3 program. The amplicon was tested by Melt Curve Analysis on ABI StepOnePlus instrument. GAPDH was used as endogenous control reference gene and all data were normalized to GAPDH expression. Primers used for the comparative C_T_ experiments were designed by the Primer Express 3.0 program (Applied Biosystems). Primer sequences are given in the supplementary material.

### Analysis of gut microbiome

Phylum-specific primer sets targeting bacterial 16S rRNA genes were used to quantitate intestinal bacteria by using DNA extracted from feces and real-time PCR^42^. 100 and 200 mg fecal content from the colon and cecum, respectively, was collected at the autopsy and frozen on dry ice. Bacterial DNA content was isolated with QIAamp DNA Stool Mini Kit (Qiagen) according the manufacturer’s instruction. Sample quality control and the quantitative analysis were carried out by NanoDrop (Thermo Scientific, USA). Quantification was done by using standard curves made from known concentrations of the respective amplicon for each set of primers. The Real-Time PCR reactions with Fast EvaGreen qPCR Master Mix (Biotium, USA) were carried out on ABI StepOnePlus instrument. The results were analyzed by ABI Step One 2.3 software. The amplicons were tested by Melt Curve Analysis on ABI Step OnePlus instrument. Copy number (CN) was calculated with the following formula: CN = A*6 × 10^23^/(L*660)*1 × 10^9^ ng/g; where A is the amount of the amplicon in ng, L is the length of the amplicon and 660 is the average MW of 1 bp double stranded DNA.

### Plasma cytokine measurement

Plasma samples were processed for cytokine measurement using Cytometric Bead Array (CBA Flex Sets BD Biosciences, UK). Eleven key cytokines/chemokines were assessed: IL-1α, IL-1β, G-CSF, IFNγ, IL-6, IL-10, IL-17A, KC, MCP-1, TNFα and RANTES (CCL5). The detection limit for each cytokine was 5–10 pg/ml.

### Statistics

Data are mean ± SEM. Statistical tests were done on GraphPad Prism 6.0 program. Multiple groups were compared by one way analysis of variance (ANOVA) and post-hoc tests between individual groups. Body weight changes were analyzed by repeated measures of ANOVA. Level of significance was set as p < 0.05.

## Additional Information

**How to cite this article**: Ferenczi, S. *et al*. Oligomannan Prebiotic Attenuates Immunological, Clinical and Behavioral Symptoms in Mouse Model of Inflammatory Bowel Disease. *Sci. Rep.* **6**, 34132; doi: 10.1038/srep34132 (2016).

## Supplementary Material

Supplementary Information
