# Supplementary material for: Oligomannan Prebiotic Attenuates Immunological, Clinical and Behavioral Symptoms in Mouse Model of Inflammatory Bowel Disease
Source: Sci Rep. 2016 Sep 23;6:34132. doi: 10.1038/srep34132 (PMC5034233; doi:10.1038/srep34132)
Supplement: Supplementary Information [file srep34132-s1.pdf]

Supplement to „Oligomannan Prebiotic Attenuates Immunological, Clinical and Behavioral Symptoms in Mouse Model of Inflammatory Bowel Disease"

by Szilamér Ferenczi[1], Krisztián Szegi[1], Zsuzsanna Winkler[1], Teréz Barna[2] and Krisztina J. Kovács[1]*

[1] Laboratory of Molecular Neuroendocrinology, Institute of Experimental Medicine, Budapest, Hungary and [2] Department of Genetics and Applied Biochemistry, Unversity of Debrecen, Debrecen, Hungary

Primer sequences used in qRT PCR reactions

GAPDH: f: TGA CGT GCC GCC TGG AGA AA, r: AGT GTA GCC CAA GAT GCC CTT CAG
IL-1b f: GCCTCGTGCTGTCGGACCCA , r: TGAGGCCCAAGGCCACAGGT
IL-1a: f: CCA TAA CCC ATG ATC TGG AAG AG, r: GCT TCA TCA GTT TGT ATC TCA AAT CAC
TNFa: f: CAG CCG ATG GGT TGT ACC TT, r: GGC AGC CTT GTG CCT TGA
MCP-1: f: CCAGCACCAGCACCAGCCAA, r: TGGATGCTCCAGCCGGCAAC
IL-6 f: CTCTGCAAGAGACTTCCATCC, r: AGTCTCCTCTCCGGACTTGT
G-CSF f: TGCCCAGAGGCGCATGAAGC, r: GGGGAACGGCCTCTCGTCCT
Arginase-1 f: GTCTGGCAGTTGGAAGCATCT, r: GCATCCACCCAAATGACACA
TLR2 f:AACCTCAGACAAAGCGTCAA, r: TCCTGAGCAGAACAGCGTTT
TLR4 f: TCCCTGCATAGAGGTAGTTCCTA, r: TTCAAGGGGTTGAAGCTCAGA
TLR7 f: TGGCTCCCTTCTCAGGATGA, r: GTGTCCACATCGAAAACACCATT
TLR9 f: GAGAGACCCTGGTGTGGAAC, r: CCTTCGACGGAGAACCATGT
NALP3 f: CAGAGCCTACAGTTGGGTGAA, r: ACGCCTACCAGGAAATCTCG
NALP6 f:CTCTCCGTGTCAGCGTTCAA, r:AGCGAGCATTCCTCTCCTTC
IL-10 f: AGTGAGAAGCTGAAGACCCTCAGG, r: TTCATGGCCTTGTAGACACCTTGGT
IL-17 f: CCTGGCGGCTACAGTGAAG, r: GGAAGTCCTTGGCCTCAGTGT
KC f: GAGCTGCGCTGTCAGTGCCT, r: CAAGGCAAGCCTCGCGACCA
CX3CL1 f:CCGCGTTCTTCCATTTGTGT, r:GGTCATCTTGTCGCACATGATT
MUC2 f: GCTGACGAGTGGTTGGTGAATG, r: GATGAGGTGGCAGACAGGAGAC
